# Bacterial inducible expression of plant cell wall-binding protein YesO through conflict between *Glycine max* and saprophytic *Bacillus subtilis*

**DOI:** 10.1038/s41598-020-75359-0

**Published:** 2020-10-29

**Authors:** Haruka Sugiura, Ayumi Nagase, Sayoko Oiki, Bunzo Mikami, Daisuke Watanabe, Wataru Hashimoto

**Affiliations:** 1 Laboratory of Basic and Applied Molecular Biotechnology, Division of Food Science and Biotechnology, Graduate School of Agriculture, Kyoto University, Uji, Kyoto 611-0011 Japan; 2 Laboratory of Applied Structural Biology, Division of Applied Life Sciences, Graduate School of Agriculture, Kyoto University, Uji, Kyoto 611-0011 Japan

**Keywords:** Microbiology, Molecular biology, Structural biology

## Abstract

Saprophytic bacteria and plants compete for limited nutrient sources. *Bacillus subtilis* grows well on steamed soybeans *Glycine max* to produce the fermented food, *natto*. Here we focus on bacterial responses in conflict between *B. subtilis* and *G. max*. *B. subtilis* cells maintained high growth rates specifically on non-germinating, dead soybean seeds. On the other hand, viable soybean seeds with germinating capability attenuated the initial growth of *B. subtilis*. Thus, *B. subtilis* cells may trigger saprophytic growth in response to the physiological status of *G. max*. Scanning electron microscope observation indicated that *B. subtilis* cells on steamed soybeans undergo morphological changes to form apertures, demonstrating cell remodeling during saprophytic growth. Further, transcriptomic analysis of *B. subtilis* revealed upregulation of the gene cluster, *yesOPQR*, in colonies growing on steamed soybeans. Recombinant YesO protein, a putative, solute-binding protein for the ATP-binding cassette transporter system, exhibited an affinity for pectin-derived oligosaccharide from plant cell wall. The crystal structure of YesO, in complex with the pectin oligosaccharide, was determined at 1.58 Å resolution. This study expands our knowledge of defensive and offensive strategies in interspecies competition, which may be promising targets for crop protection and fermented food production.

## Introduction

Plant seeds, each consisting of an embryo, storage tissues, and protective outer coat, remain dormant, waiting for favorable environmental conditions for germination. Stored nutrient sources, such as carbohydrates, lipids, and proteins, are available to the germinating embryo, and also to seed microbiota, a community of bacteria and fungi surrounding the seeds^1,2^. Plants have evolved sophisticated mechanisms to prevent phytopathogen colonization and infection. However, once ungerminated seeds undergo senescence, decreased antimicrobial activity of dead seeds and, thus, increased accessibility to nutrients may permit microbial growth using seed decomposition products, i.e., saprophytic growth^3–5^. The molecular basis for recognition of seed physiological status is required to understand the competition between microorganisms and plants.

*Bacillus subtilis* is a Gram-positive, spore-forming, saprophytic bacterium ubiquitously isolated from soil, water, air, decaying plant materials, and fermented foods^6–9^. During production of *natto*, a traditional fermented soybean food in Japan, soybean seeds are soaked and steamed before inoculation with *B. subtilis*. This process, which kills seeds, is regarded as essential for enhancing *B. subtilis* growth. Therefore, *B. subtilis* cells growing on soybean seeds may be a useful model to study cell responses and molecular recognition at saprophytic growth onset. *B. subtilis* is widely used in emerging research fields as a Gram-positive model organism^10,11^; however, its physiological responses and behaviors on soybean seeds remain unclear.

Saprophytic growth requires nutrient uptake by decomposer organisms from decaying plant material. Bacterial ATP-binding cassette (ABC)-family transporters translocate a variety of substrates into cells, using energy from ATP hydrolysis^12–14^. A typical ABC transporter consists of three components: integral membrane proteins that form pores, nucleotide-binding proteins (NBPs) that bind and hydrolyze ATP, and solute-binding proteins (SBPs) that recognize and deliver target compounds into cells. SBPs are critical elements for substrate specificity and high affinity of ABC transporter systems^15–18^. For instance, a conformational change of SBP in pathogenic bacteria is critical for selective recognition and import of mammalian host glycosaminoglycans^19,20^.

Pathogenic and symbiotic microorganisms secrete numerous enzymes to penetrate and degrade plant cell walls to provide nutrition^21–25^. Thus, cell wall decomposition products are potential targets for saprophytic bacteria, which may use them to monitor plant physiological status. Primary plant cell walls consist of a cellulose-hemicellulose framework embedded in an inter-fibrillar matrix of pectins^26,27^. Complex and diverse pectic polysaccharides include homogalacturonan (HG), rhamnogalacturonan type I (RG-I), and rhamnogalacturonan type II (RG-II). HG is a linear polymer of α-1,4-linked galacturonic acid. RG-I is composed of a backbone, based on disaccharide-repeating units of rhamnose and galacturonic acid, with side chains of galactans, arabinans, and arabinogalactans. RG-II contains HG as a backbone, with complex side chains composed of a variety of sugars. Although studies on HG degradation have progressed^28–30^, bacterial decomposition of RG-I and RG-II has been reported in a limited number of papers so far^31–33^.

This report is the first on the competition between saprophytic *B. subtilis* and soybean *Glycine max* on the surfaces of soybean seeds. This article further focuses on the physiological and transcriptomic traits of *B. subtilis*, and provides structural insight into SBP-dependent recognition of RG-I-derived trisaccharide during the saprophytic growth.

## Results and discussion

### Growth of *B. subtilis* on live and dead soybeans

*Natto*, a traditional Japanese fermented food, is manufactured from steamed soybean seeds^7,9^. Saprophytic *B. subtilis* cells grow on dead soybeans, using decomposition products as nutrients. Steaming may cause not only loss of viability but also denaturation of proteins; it may alter other soybean components as well. Therefore, what exactly triggers saprophytic growth is an open question. Heat-related impacts to soybean components were reduced by adopting physiological senescence, induced at different temperatures, by prolonged seed storage. Six-month storage at 4 °C maintained viability, but six-month storage at an ambient temperature severely limited seed germination (Supplementary Fig. S1A). Thus, seeds stored under the former and the latter conditions were used as live and dead soybeans, respectively.

**Figure 1:**
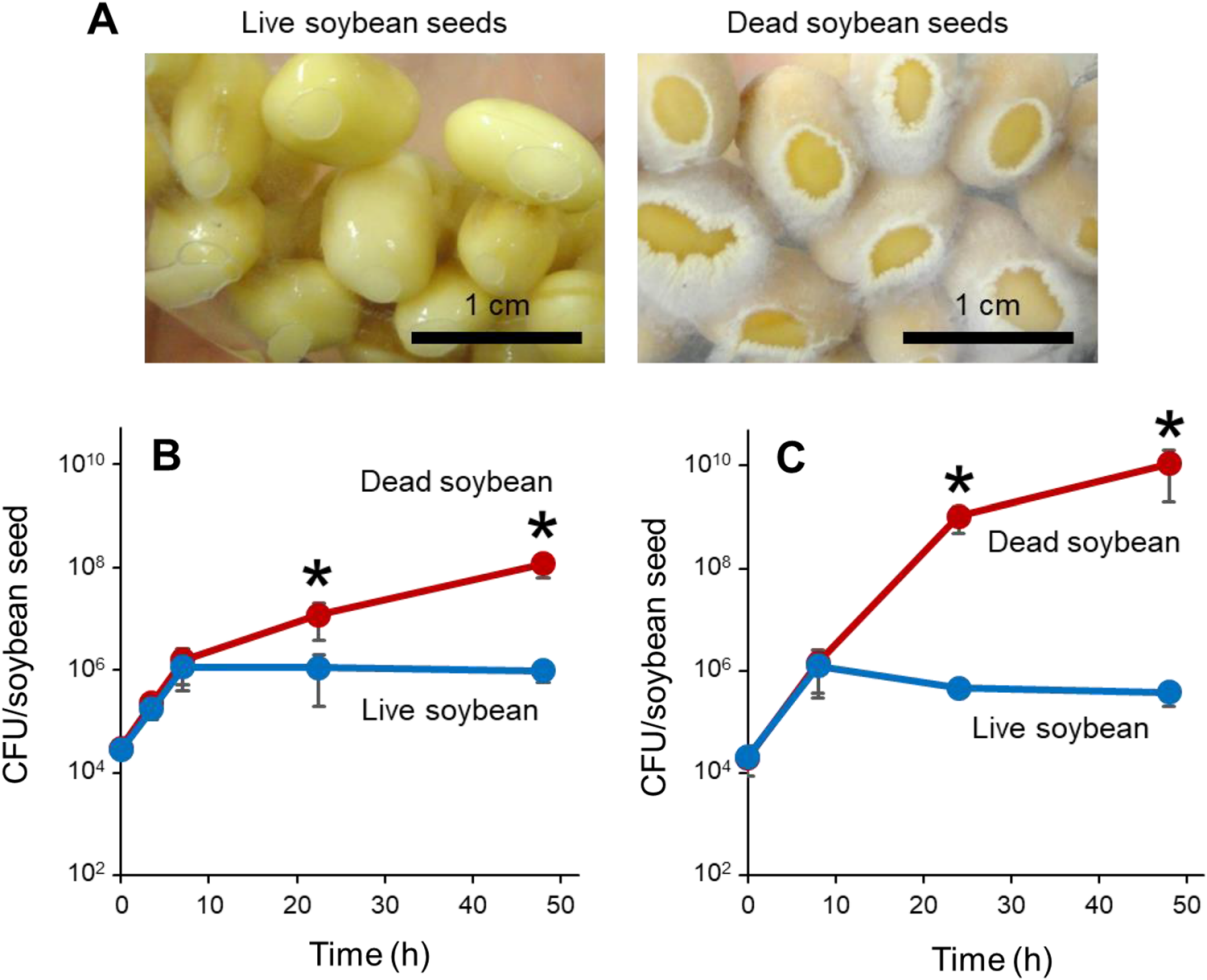
Saprophytic growth of *B. subtilis* on soybean surfaces. (**A**) Soybean seeds inoculated with *B. subtilis* NBRC 16449 cells at 24 h after inoculation. Live (left) or dead (right) soybeans were used. Bars, 1 cm. (**B**) Growth curves of *B. subtilis* NBRC 16449 cells on soybean seeds. (**C**) Growth curves of *B. subtilis* 168 cells on soybean seeds. Each data point represents the mean ± standard deviations (SD) from three independent experiments. Asterisks indicate significant differences from the data of live soybeans (*p* < 0.05; *t*-test)

*Bacillus subtilis* cells were found to grow vigorously on non-germinating dead soybean seeds, but not on live seeds (Supplementary Video S1, Fig. 1A). Thus, seed germination and bacterial growth inhibition may be closely linked. We first discovered this phenomenon using *B. subtilis natto*-producing strain NBRC 16449^34^. Quantitative analysis of colony-forming units (CFUs) indicated that *B. subtilis* initially proliferated on both live and dead soybeans. However, growth attenuated specifically on live soybeans when bacterial cell numbers reached 10^6^ CFUs per seed (Fig. 1B). *B. subtilis* laboratory standard strain 168^35^ exhibited a similar growth profile on live and dead seeds (Fig. 1C). Thus, live soybeans can severely limit *B. subtilis* growth on their surfaces. Invading bacteria and germinating plant embryos may compete for nutrients in soybean seeds. *B. subtilis* cells gain access to seed nutrients when the seeds lose germination ability. *B. subtilis* growth on soybean seeds is a promising model for bacterial-plant interactions in seed microbiota.

### Morphological traits of *B. subtilis* during saprophytic growth

**Figure 2:**
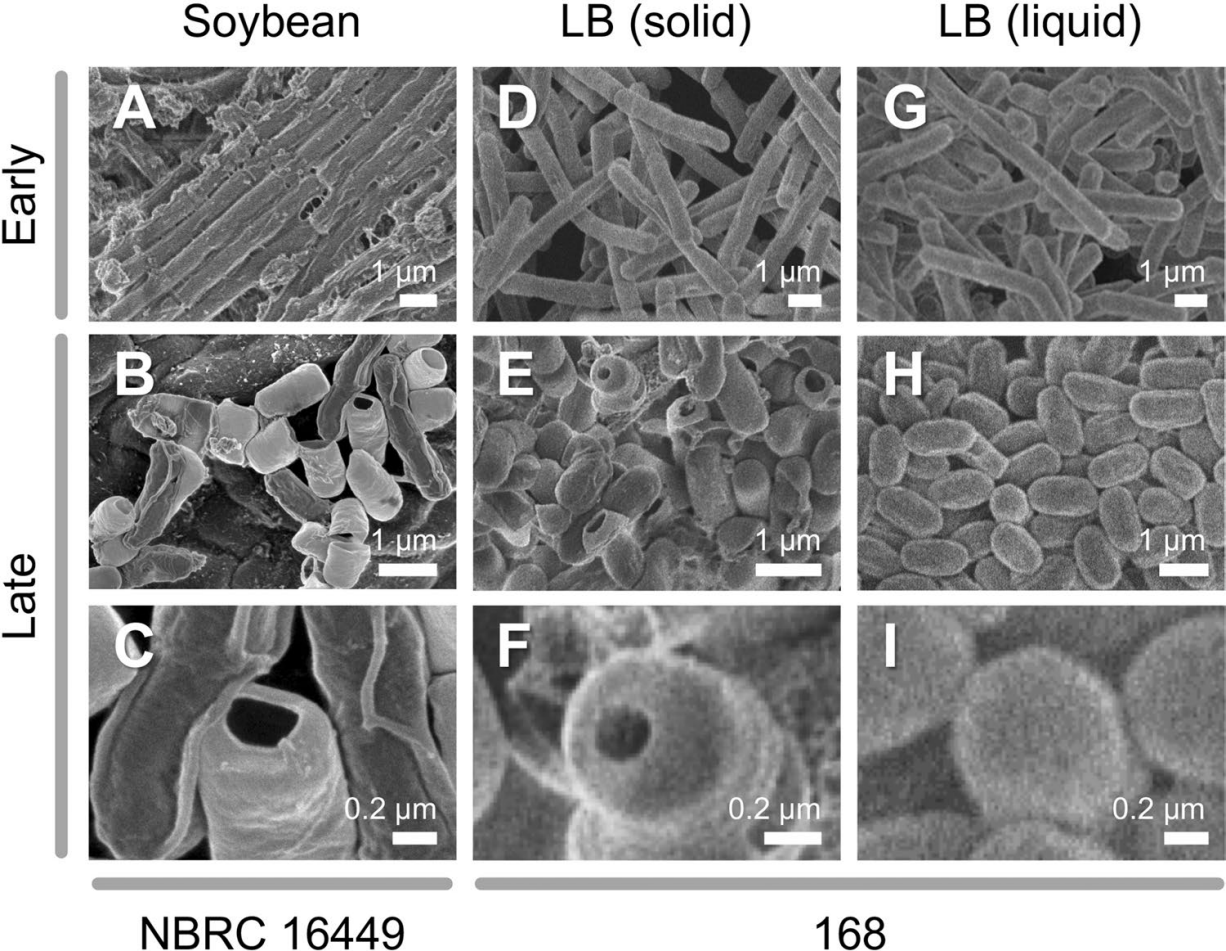
SEM observation of *B. subtilis* cell morphology. (**A**) *B. subtilis* NBRC 16449 cells, 5 h after inoculation on steamed soybean seeds. (**B**,**C**) *B. subtilis* NBRC 16449 cells, 48 h after inoculation on steamed soybean seeds. (**D**) *B. subtilis* 168 cells, 4 h after inoculation on soybean-mimicking solid medium blocks. (**E**,**F**) *B. subtilis* 168 cells, 216 h after inoculation on soybean-mimicking solid medium blocks. (**G**) *B. subtilis* 168 cells, 4 h after inoculation in liquid medium. (**H**, **I**) *B. subtilis* 168 cells, 239 h after inoculation in liquid medium. Bars, 1 μm (**A**,**B**,**D**,**E**,**G**,**H**) or 0.2 μm (**C**,**F**,**I**).

To understand the strategy of *B. subtilis* for saprophytic growth on dead soybeans during *natto* production, morphological and transcriptional responses were examined. Scanning electron microscope (SEM) analysis revealed cell morphological changes of *B. subtilis* NBRC 16449 during saprophytic growth on steamed seeds (Fig. 2A–C). The early logarithmic growth phase, 5 h after inoculation, was characterized by elongated, rod-shaped cells, 6–10 μm long. As the growth progressed, most cells displayed short-rod forms, approximately 2 μm long, with distinctive aperture-like structures (Fig. 2C). *B. subtilis* NBRC 16449 (Supplementary Fig. S2) and 168 (Fig. 2D–F) strains exhibited apertures on soybean seed-mimicking solid medium (Supplementary Fig. S1B). No apertures were observed in liquid medium (Fig. 2G–I), suggesting that a solid substrate is needed for this culture-specific response. Similar aperture-like structures are previously reported in *Clostridium sporogenes* during formation of endospores^36^. *B. subtilis* cell membranes are subject to engulfment during spore development under nutrient-depleted conditions^37,38^, and saprophytically growing *B. subtilis* may undergo similar morphological remodeling. How solid-state culture stimulates the starvation-triggered sporulation pathway is of great interest from a signal transduction viewpoint.

### Transcriptomic profile of *B. subtilis* during saprophytic growth

**Figure 3:**
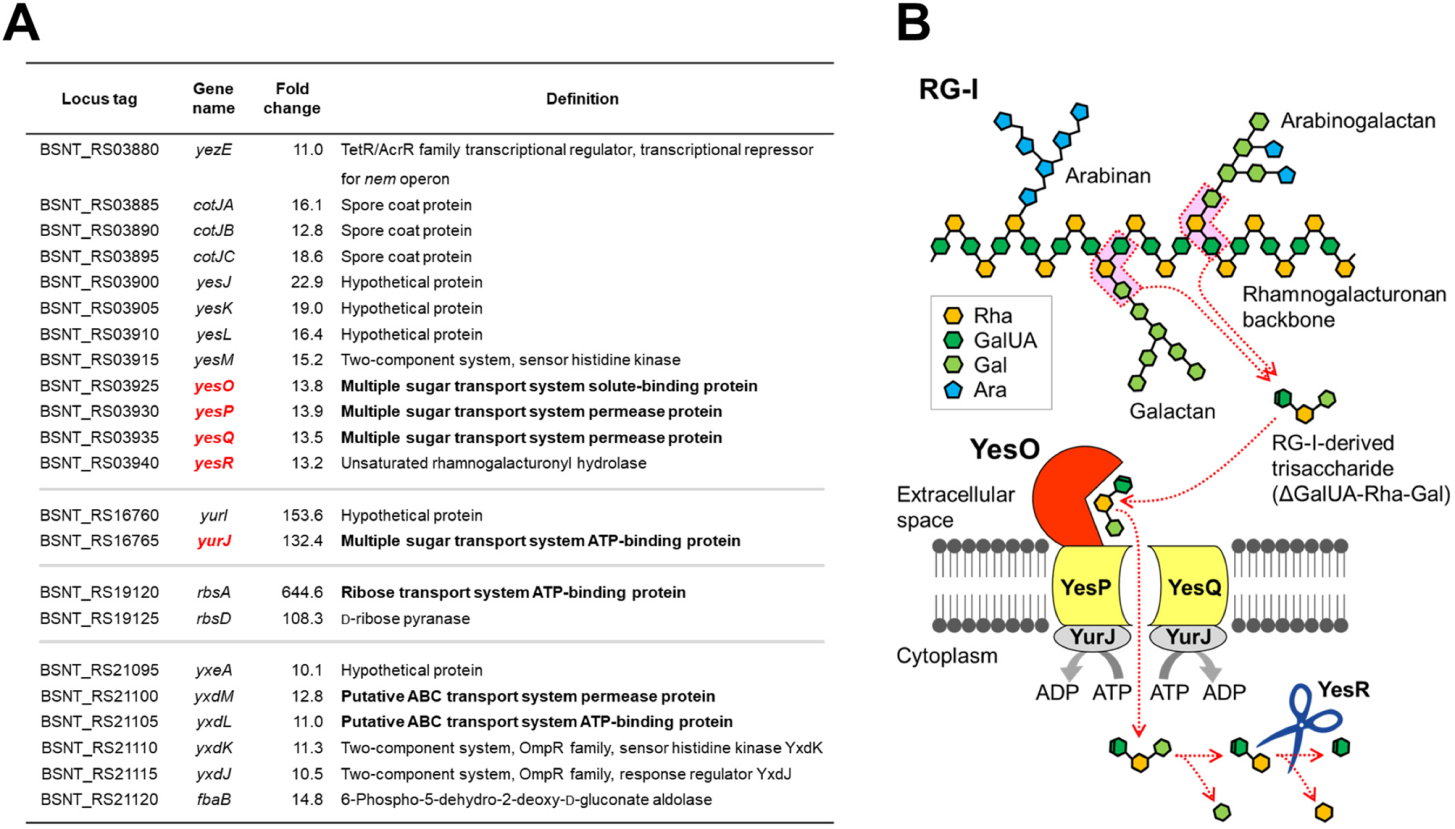
Transcriptional response of *yesOPQR* and *yurJ* genes during saprophytic growth. (**A**) ABC transporter component genes and neighboring genes upregulated during saprophytic growth on dead soybeans (over tenfold). (**B**) Model of YesOPQR/YurJ-dependent incorporation of RG-I-derived trisaccharide.

Comprehensive understanding of saprophytism-specific recognition and responses was sought using the transcriptomic profiles of *B. subtilis* NBRC 16449 cells from steamed soybeans and from soybean seed-mimicking solid medium, using RNA-Seq analysis. Reads were mapped to the genomic sequence of *B. subtilis* subsp. *natto* BEST195 strain^39,40^. Expression of 93 and 107 genes among a total of 4271 analyzed genes was over tenfold upregulated and downregulated, respectively, in *B. subtilis* grown on steamed soybeans. Upregulated transcripts included several ABC transporter components and their neighboring genes (Fig. 3A). The gene cluster *yesOPQR* was highly expressed, as previously reported in *B. subtilis* 168 cells grown on RG-I, a component of plant cell wall pectin^32^. Based on sequence homology, *yesO* and *yesPQ* genes encode a putative SBP and transmembrane permeases for the ABC-family sugar transport system, respectively. Deleting *yesOPQ* has a negative impact on utilization of RG-I as a carbon source^41^. YesR is a cytosolic hydrolase that acts on unsaturated rhamnogalacturonan disaccharide derived from RG-I^31^. Another upregulated gene, *yurJ*, may encode a functional NBP for YesOPQ and other ABC transporters^41,42^. Thus, *B. subtilis* may recognize and use plant cell wall-derived RG-I as a carbon source by inducing *yesOPQR* and *yurJ* genes during saprophytic growth (Fig. 3B).

### Affinity between YesO and RG-I oligosaccharides

**Figure 4:**
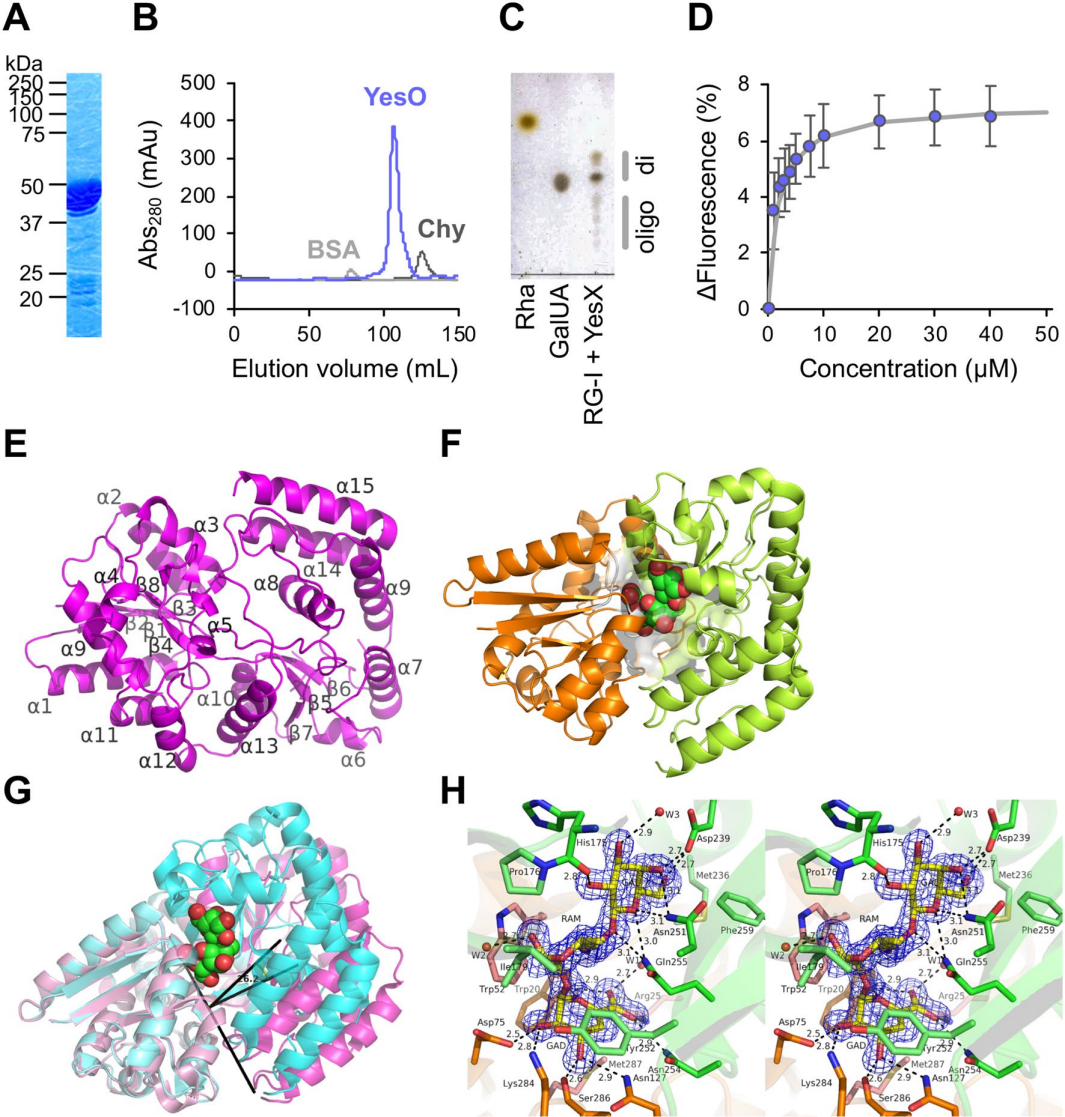
Interaction between oligo-RG-I and YesO. (**A**) SDS-PAGE analysis of purified YesO, followed by CBB staining. (**B**) Elution profile of YesO via gel filtration chromatography. Bovine serum albumin (BSA, 66 kDa, light gray) and chymotrypsin (Chy, 25 kDa, dark gray) were used for comparison. (**C**) TLC analysis of oligo-RG-I. Note that unsaturated rhamnogalacturonan disaccharide (di) showed two distinctive signals, corresponding to ammonium ion-unbound and bound forms^32^. Traces of oligosaccharides (oligo) were also detected. Rhamnose (Rha) and galacturonate (GalUA) were used for comparison. The profile of TLC plate is not an image cropped from different parts of the same plate or from different plates. (**D**) Fluorescent spectrum analysis. The decrease in fluorescent intensity (Δfluorescence), caused by increasing ligand concentrations, was plotted after modification based on volume change in the cuvette. Each data point represents the mean ± SD from three independent experiments. (**E**) Overall structure of ligand-free YesO protein. The assigned secondary structures of α-helices (α1–α15) and β-sheets (β1–β8) are indicated. (**F**) Overall structure of YesO/oligo-RG-I. Orange, N-domain; green, C-domain. (**G**) Superimposition of YesO and YesO/oligo-RG-I. Magenta, YesO; cyan, YesO/oligo-RG-I. The ball model shows ΔGalUA-Rha-Gal (green, carbon atom; red, oxygen atom). The rotation axis and angle for the Venus-flytrap conformational change are shown. (**H**) Residues interacting with ΔGalUA-Rha-Gal via hydrogen bonds and C–C contacts. Electron density map (2*F*_o_–*F*_c_) of ΔGalUA-Rha-Gal in YesO/oligo-RG-I is shown in blue mesh contoured with 1.4 σ. Hydrogen bonds are represented by dotted lines with labeled distances. The residues of the N- and C-domains are colored orange and green, respectively. The residues interacting only by C–C contacts (Trp52, Pro176, Ile179, Phe180, Met236, Tyr252, Phe259, and Met287) are pale-colored.

YesO is a putative SBP involved in RG-I assimilation, but its binding substrate is unknown. Recombinant YesO protein and the decomposition product of RG-I backbones (oligo-RG-I) were prepared to address this knowledge gap. Comparisons between YesO of *B. subtilis* laboratory strain 168 and its orthologs from *Bacillus sonorensis*, *Bacillus licheniformis* DSM 13, *Paenibacillus* sp. Aloe-11, and *Paenibacillus macerans* revealed low conservation of the N-terminus (Supplementary Fig. S3). Thus, N-terminus-deleted YesO, of approximately 45 kDa, was overexpressed in *Escherichia coli*, and purified to homogeneity (Fig. 4A,B). To obtain oligo-RG-I, RG-I backbones were treated with exolyase YesX (Fig. 4C). Thin-layer chromatography (TLC) analysis of reaction products showed that released oligosaccharides consisted mainly of unsaturated rhamnogalacturonan disaccharide, as reported previously^32^. The interaction between YesO and oligo-RG-I was analyzed by measuring YesO fluorescent intensity in the presence or absence of oligo-RG-I (Fig. 4D). Adding oligo-RG-I decreased the fluorescent intensity from tryptophan residues of YesO by 3–7%. Based on the plot, the dissociation constant (*K*_d_) was 1.6 μM. No significant decrease of fluorescent intensity was observed when other disaccharides, such as sucrose, maltose, cellobiose, chitobiose, and digalacturonic acid, were used as a ligand (Supplementary Fig. S4). YesO appears to recognize oligo-RG-I as a substrate selectively.

### Binding mode of YesO to oligo-RG-I

Oligo-RG-I recognition by YesO was analyzed with X-ray crystallography. The crystal structures of YesO and its complex form with oligo-RG-I (YesO/oligo-RG-I) were determined at 1.97 Å and 1.58 Å resolution, respectively. Data collection and refinement statistics are summarized in Supplementary Table S1. Ligand-free YesO structure (Fig. 4E) was similar to a previously determined structure (PDB ID 4R6K). YesO is an α/β protein with 15 α helices (residues 23–39, 52–65, 76–86, 92–96, 111–113, 135–140, 152–166, 177–187, 205–219, 253–262, 312–318, 328–336, 340–354, 368–383, and 381–405) and 8 β sheets (residues 15–20, 45–55, 71–73, 122–126, 130–133, 248–252, 267–270, and 288–290). Note that amino acid residue numbers of our YesO are 20 fewer than those of the full-length YesO to adjust to PDB ID 4R6K. The substrate-binding cleft is formed between two globular N- and C-domains (Fig. 4F), as typically observed among SBPs of ABC transport systems^15–18^. YesO is categorized as a Class II SBP, based on a β_2_–β_1_–β_3_–β_8_–β_4_ topology of the β sheet core in the N-domain. N- and C-domains are connected through two short hinges (residues Val126-Leu129 and Leu281-Lys284), assigning YesO to Cluster D SBPs. Comparison of ligand-free YesO with YesO/oligo-RG-I revealed a 26.2°-closed conformation upon binding of a ligand molecule (Fig. 4G), suggesting a Venus-flytrap conformational change^43^.

Although our prepared oligo-RG-I was mostly disaccharide (Fig. 4C), X-ray crystallography of YesO/oligo-RG-I indicated an interaction between YesO and the trisaccharide, ΔGalUA-Rha-Gal, composed of unsaturated galacturonic acid and rhamnose from the main chain and galactose from the side chain (Fig. 4H). This result reproduced another independent YesO/oligo-RG-I crystal structure. The molecular docking simulation suggested that rhamnogalacturonan tetrasaccharide is too large to fit in the ligand-binding cleft (Supplementary Fig. S5). Thus, YesO may selectively recognize ΔGalUA-Rha-Gal trisaccharide as a ligand. ΔGalUA-Rha-Gal binds to the YesO cleft via hydrogen bonds and C–C contacts (Table 1). There were 19 hydrogen bonds (ΔGalUA, 10; Rha, 2; Gal, 7) and 44 C–C contacts (ΔGalUA, 22; Rha, 17; Gal, 5). ΔGalUA-interacting residues, Arg25, Asn127, Asn254, and Ser286, are highly conserved among the known YesO orthologs, highlighting the importance of ΔGalUA in the YesO ligand.

**Table 1:** Interaction between YesO and ΔGalUA-Rha-Gal.

Hydrogen bond (< 3.3 Å)					van der Waals interaction (< 4.5 Å)				
Source		Target		Distance (Å)	Source		Target		Distance (Å)
Sugar	Atom	Protein/water	Atom		Sugar	Atom	Protein	Atom	
ΔGalUA	O2	Asp75	OD1	2.5	ΔGalUA	C1	Trp20	CE2	4.3
		Lys284	NZ	2.8			Trp20	CZ2	3.9
	O3	Ser286	O	2.6			Trp52	CE3	3.9
		Asn127	ND2	2.9			Trp52	CZ3	3.6
	O5	Trp20	NE1	3.1			Trp52	CH2	4.1
	O6A	Arg25	NH2	2.8		C2	Trp20	CZ2	4.2
		Asn254	ND2	2.9			Trp52	CZ3	4.4
	O6B	Trp20	NE1	2.9			Asp75	CG	4.2
		Arg25	NH1	3.0		C3	Tyr252	CG	4.2
		water631-Gln255	NE2	2.7, 3.2			Tyr252	CD2	3.5
							Tyr252	CE2	3.7
							Lys284	CE	4.2
						C4	Met287	CE	4.3
							Gln255	CD	4.4
							Tyr252	CD2	4.0
						C5	Trp20	CZ2	4.3
							Met287	CE	4.1
							Gln255	CD	4.3
						C6	Arg25	CZ	4.0
							Met287	CE	3.8
							Gln255	CD	4.1
							Gln255	CG	4.1
Rha	O1	water634-Asp178	OD1	2,7, 2.8	Rha	C1	Trp52	CE2	4.4
	O4	Gln255	NE2	3.1			Trp52	CZ2	4.2
							Ile179	CG1	4.1
						C2	Trp52	CD2	4.0
							Trp52	CE2	4.1
							Trp52	CE3	4.0
							Trp52	CZ2	4.2
							Trp52	CZ3	4.0
							Trp52	CH2	4.1
						C4	Gln255	CD	3.9
						C5	Pro176	CD	3.9
							Tyr252	CE2	4.2
						C6	Pro176	CD	4.2
							Tyr252	CD2	4.0
							Tyr252	CE2	3.9
							Phe180	CE2	3.9
							Phe180	CZ	4.0
Gal	O2	His175	O	2.8	Gal	C3	His175	CD2	4.1
	O3	water659-Asn172	ND2	2.9, 3.0		C4	Asp239	CG	4.1
		water659-Asp239	OD1	2.9, 2.7		C6	Met236	CE	3.9
	O4	Asp239	OD2	2.7			Phe259	CE1	4.0
		Asn251	ND2	3.1			Phe259	CZ	3.8
	O5	Asn251	ND2	3.1					
		Gln255	NE2	3.1					
	O6	Asp239	OD2	2.7					

### Recognition of ΔGalUA-Rha-Gal during saprophytic growth

In the present study, *B. subtilis yesOPQR* was identified as an upregulated gene cluster during saprophytic growth on dead soybean surfaces (Fig. 3B). YesO was found to act as an SBP for uptake of RG-I-derived ΔGalUA-Rha-Gal trisaccharide. YesPQ are putative transmembrane permeases, and YesR catalyzes intracellular degradation of unsaturated rhamnogalacturonan disaccharide^31^. Expression of the *yurJ* gene, encoding a putative NBP for YesOPQ^40^, was also induced on dead soybeans. Thus, YesOPQR/YurJ-dependent assimilation of ΔGalUA-Rha-Gal is likely to play a major role in saprophytic growth. Assuming that plant cell wall degradation is closely associated with wounding, pathogen attack, or cell death, induction of the *yesOPQR* and *yurJ* genes might be one of the initial responses for saprophytic growth on dead plant surfaces.

Our hypothesis is that ΔGalUA-Rha-Gal is used as a nutrient or signaling molecule, or both. ΔGalUA-Rha-Gal is degraded into assimilable monosaccharides by an unidentified intracellular β-1,4-galactosidase and YesR. Since the soybean surface is a low-nutrient environment, pectin-derived oligosaccharides may be the carbon source that supports saprophytic growth. Further, ΔGalUA-Rha-Gal may also serve as a signaling molecule to trigger saprophytism. Some bacterial two-component regulatory systems adopt SBPs for signal recognition^18^. Our recent study demonstrated that SPH1118, an SBP in *Sphingomonas* sp. strain A1, is responsible not only for pectin assimilation but also for chemotaxis toward pectin^44^. Assuming such alternative functions of YesO, recognizing ΔGalUA-Rha-Gal may trigger signal transduction for proliferation or adaptation under saprophytic conditions.

Based on the YesO/oligo-RG-I three-dimensional structure, YesO traps ΔGalUA-Rha-Gal trisaccharide, although most of the oligo-RG-I used in this study was disaccharide. How is ΔGalUA-Rha-Gal generated via pectin degradation? *B. subtilis* cells secrete a series of enzymes for RG-I degradation, such as YesWX lyases to cleave α-1,4-glycosidic linkages between rhamnose and galacturonic acid residues in the RG-I backbone^32^, GanAB galactosidases to degrade galactan branches^45^, and AbfA and Xsa arabinofuranosidases to catalyze hydrolysis of terminal arabinofuranoside bonds of arabinogalactan or arabinan^46^. YesWX-dependent degradation of RG-I backbones, with a single galactose residue at each branch point, may release ΔGalUA-Rha-Gal trisaccharide (see red areas in Fig. 3B).

In conclusion, this report is the first to describe the competition between saprophytic *B. subtilis* and soybean *G. max* on soybean seeds surfaces. Live soybeans prevent bacterial invasion, while *B. subtilis* YesO recognizes pectin-derived ΔGalUA-Rha-Gal trisaccharide to establish saprophytic growth on dead soybean seeds. This study will help elucidate the diversity of bacterial-plant interactions, particularly for seed microbiota.

## Materials and methods

### Materials and microorganisms

Soybean seeds of Suzumaru (*G. max*) were purchased from Mamehei (Japan). RG-I (from potatoes) was purchased from Megazyme (Ireland). *B. subtilis* laboratory standard strain 168 was obtained from the National Bio Resource Project (NBRP), Japan. *B. subtilis* strain NBRC 16449 was obtained from the NITE Biological Research Center (NBRC), Japan. *E. coli* strain BL21-Gold(DE3) (Agilent, USA) was used for recombinant protein overexpression. *E. coli* strain DH5α was used for cloning and plasmid maintenance. Bacterial cells were routinely grown in LB medium (1% tryptone, 0.5% yeast extract, 1% sodium chloride, pH 7.2) at 37 °C.

### Growth test

Soybean seeds were stored at 4 °C and at room temperature for 6 months to prepare live and dead soybeans, respectively. The seeds were washed and soaked in pure water at room temperature for 3.5 h. The seed surfaces were sterilized by 20-min ultraviolet exposure. This exposure did not produce severe effects on living soybean seeds’ germination capability (Supplementary Fig. S1A). Soybean seed-mimicking solid LB medium blocks (solidified by 3% agar, 300 μl each; Supplementary Fig. S1B) were prepared aseptically in microtubes. Thirty seeds or blocks were used for each growth test.

*Bacillus subtilis* was precultured at 37 °C in liquid LB medium overnight, washed, and resuspended in 0.85% sodium chloride. Approximately 10^4^ cells per seed or solid medium block were inoculated and incubated at 37 °C. To assay CFUs, *B. subtilis* cells on the sampled seeds or blocks were collected in 0.85% sodium chloride, spread on LB agar plates, and incubated at 37 °C overnight.

### SEM analysis

*Bacillus subtilis* cells were grown on steamed soybean seeds, on soybean mimicking solid LB medium blocks, and in liquid LB medium at 37 °C. Steamed soybeans were prepared by washing and soaking live seeds with pure water at room temperature for 3.5 h, followed by autoclaving at 121 °C for 20 min. For protein fixation, samples (soybean seeds or collected cells) were mixed with 4% formaldehyde and incubated for 1 h. Subsequently, for lipid fixation, samples were washed with 10 mM potassium phosphate buffer or pure water three times and immersed in 1% osmium oxide for 1–2 h. Fixed samples were washed with 10 mM potassium phosphate buffer or pure water three times, dehydrated sequentially with 50%, 70%, 90%, and 100% ethanol, and immersed in *t*-butyl alcohol at 4 °C. Freeze-dried samples were coated with a platinum-palladium layer under argon gas and observed with a field emission (FE)-SEM SU8230 (Hitachi, Japan) using a 1.5 kV electron beam.

### RNA-Seq analysis

*Bacillus subtilis* NBRC 16449 cells were grown on steamed soybean seeds or soybean-mimicking solid LB medium blocks to the logarithmic phase. Cells on the seeds or blocks were collected in 0.85% sodium chloride, treated with RNAprotect bacteria reagent (Qiagen, Netherlands), and frozen immediately in liquid nitrogen. Total RNA was prepared using the hot phenol method^47^ by Nihon Gene Research Laboratories, Inc. (Japan). RNA-Seq analysis was performed by Macrogen Japan Corp. (Japan). In brief, paired-end sequencing of the constructed library was performed using HiSeq 2500 (Illumina, USA). Raw data were filtered using Trimmomatic v0.32^48^ to remove adapter sequences and bases with a Phred score below 30. Trimmed reads were aligned against the *B. subtilis* subsp. *natto* BEST195 reference genome (DDBJ accession no. DRA000001)^39,40^, using Bowtie as the alignment algorithm^49^. Differential expression analysis was conducted using HTSeq^50^. RNA-Seq data, generated from this study, were deposited in the NCBI Gene Expression Omnibus (GEO) and are accessible through GEO series accession number GSE109523.

### Expression and purification of recombinant YesO protein

To construct an expression system for YesO that lacks the weakly conserved N-terminus (Supplementary Fig. S3), the *yesO* gene of *B. subtilis* strain 168 was amplified by genomic PCR, using primers 5′-GGCATATGACACTCAGAATCGCGTGGTGGGGC-3′ and 5′GGCTCGAGTCAATTATTCCTCTCTAATATCTCATT-3′ (with underlined restriction sites), and cloned into the NdeI-XhoI site of pET21b(+) (Novagen, USA). The resultant pET21b(+)-*yesO* plasmid was introduced into *E. coli* BL21-Gold(DE3) cells.

*Escherichia coli* cells harboring the plasmid were cultured in LB liquid medium with 100 μg ml^−1^ ampicillin at 30 °C. When optical density at 600 nm (OD_600_) reached 0.3, isopropyl-β-d-thiogalactopyranoside (IPTG) was added to the culture at a final concentration of 0.1 mM. After further incubation at 16 °C for 2 days, cells were washed, suspended in 20 mM Tris–HCl (pH 7.5), and disrupted with an ultrasonicator 201 M (Kubota, Japan). The supernatant obtained by centrifugation (20,000 × *g*, 4 °C, 20 min) was used for YesO purification.

Cell extracts were applied to an anion exchange chromatography system with a TOYOPEARL DEAE-650M column (Tosoh, Japan). Proteins were eluted using a linear gradient of sodium chloride (0–250 mM) in 20 mM Tris–HCl (pH 7.5). After separation with 12.5% SDS-PAGE, fractions containing the target protein were combined and purified by gel filtration chromatography using HiLoad 16/60 Superdex 200 pg resin (GE Healthcare, USA) equilibrated with 200 mM sodium chloride in 20 mM Tris–HCl (pH 7.5). After separation with 12.5% SDS-PAGE, fractions containing the target protein were combined.

### Preparation of oligo-RG-I

Substrate RG-I backbones were prepared from commercially available RG-I, as previously reported^32^. A reaction mixture, consisting of 1% RG-I backbone, 50 mM Tris–HCl (pH 7.5), 20 mM manganese(II) chloride, and lysates of YesX-expressing *E. coli* cells^32^, was incubated at 30 °C for 3 h. The mixture was boiled for 5 min and centrifuged (7200 × *g*, room temperature, 5 min). The supernatant was passed through a Centriprep centrifugal filter (3 kDa NMWL; Merck Millipore, USA) to obtain decomposition products. Resultant oligo-RG-I was detected by TLC analysis, as reported previously^32^.

### Fluorescence spectrum analysis

Fluorescence of protein, with a peak around 340 nm, is partially quenched by adding a binding ligand^51^. Fluorescent intensities of YesO, with increasing concentrations of oligo-RG-I, were measured by spectrophotometer FP-6500 (JASCO, Japan). Measurement parameters during reactions were: excitation band width, 1 nm; emission band width, 10 nm; response, 2 s; sensitivity, high; excitation wavelength, 280 nm; start to end emission wavelength, 300–500 nm; data pitch, 1 nm; and scan speed, 100 nm min^−1^. The reaction mixture contained 0.14 μM YesO, 20 mM Tris–HCl (pH 7.5), and 0–40 μM ligands. Decreases in fluorescent intensity by adding ligands were plotted, and the dissociation constant (*K*_d_) was determined based on nonlinear regression with one site-specific binding model^51^.

### X-ray crystallography

Ligand-free YesO and YesO/oligo-RG-I were crystallized by sitting drop vapor diffusion, using the JBScreen crystallization kit (Jena Bioscience, Germany). In 96-well sitting drop plates, 1 μl of 15 mg ml^−1^ YesO (without or with 1 mM oligo-RG-I) and 1 μl of reservoir solution were mixed. The mixture was kept at 20 °C until crystals grew sufficiently. Ligand-free YesO was crystallized in the drop, consisting of 100 mM malonate-imidazole-boric acid (MIB) buffer (pH 6.0), 25% polyethyleneglycol (PEG)-1500, and 1 mM digalacturonate. YesO/oligo-RG-I was crystallized in the drop, consisting of 100 mM Tris–HCl (pH 8.5), 20% PEG-1000, and 25% glycerol.

Each single crystal was picked up using a nylon loop from the drop, soaked in a reservoir solution containing 20% ethylene glycol, and instantly frozen using cold nitrogen gas. Synchroton radiation X-ray irradiated the crystals at 1.00 Å wavelength, and X-ray diffraction data were collected using a MAR225HE CCD detector (Rayonix, USA) at the BL-26B1 and BL-38B1 beamlines in SPring-8 (Japan). Diffraction data were processed using the HKL-2000 program^52^.

The crystal structures of YesO and YesO/oligo-RG-I were determined through molecular replacement with the Molrep program^53^ in the CCP4Interface package, using the ligand-free YesO structure (PDB ID 4R6K) as a reference model. Structure refinement was conducted using the phenix.refine^54^ and REFMAC5^53^ programs. At each refinement cycle, the model was adjusted manually with the WinCoot program^55^. Images of protein structures were prepared using the PyMOL molecular graphics system (Schrödinger, USA). The coordinates and structure factors (accession codes 5Z6C for ligand-free YesO and 5Z6B for YesO/oligo-RG-I) have been deposited in the PDB.

## Supplementary information

Supplementary Information 1.

Supplementary Video 1.

Supplementary Video S1.

## Data Availability

*Accession codes* RNA-Seq data were deposited in the NCBI GEO and are accessible through GEO series accession number GSE109523. Protein structures of YesO and YesO/oligo-RG-I were deposited in the PDB under accession codes 5Z6C and 5Z6B, respectively.
