# Supplementary material for: Bacterial inducible expression of plant cell wall-binding protein YesO through conflict between Glycine max and saprophytic Bacillus subtilis
Source: Sci Rep. 2020 Oct 29;10:18691. doi: 10.1038/s41598-020-75359-0 (PMC7596534; doi:10.1038/s41598-020-75359-0)
Supplement: Supplementary file 1 — Supplementary Information 1. [file 41598_2020_75359_MOESM1_ESM.pdf]

Bacterial inducible expression of plant cell wall-binding protein YesO in response to soybean: Conflict between *Glycine max* and saprophytic *Bacillus subtilis*

Haruka Sugiura[1], Ayumi Nagase[1], Sayoko Oiki[1], Bunzo Mikami[2], Daisuke Watanabe[1] & Wataru Hashimoto[1*]

[1]Laboratory of Basic and Applied Molecular Biotechnology, Division of Food Science and Biotechnology, Graduate School of Agriculture, Kyoto University, Uji, Kyoto 611-0011, Japan. [2]Laboratory of Applied Structural Biology, Division of Applied Life Sciences, Graduate School of Agriculture, Kyoto University, Uji, Kyoto 611-0011, Japan.

[*]email: hashimoto.wataru.8c@kyoto-u.ac.jp

Supplementary Information

Supplementary Figure S1. Materials used for solid-state cultures in this study.

Supplementary Figure S2. SEM observation of *B. subtilis* NBRC 16449 cells on soybean-mimicking solid medium blocks.

Supplementary Figure S3. Alignment of YesO orthologs.

Supplementary Figure S4. Fluorescent spectrum analysis.

Supplementary Figure S5. The stereo diagram of the cavity of YesO/oligo-RG-I.

Supplementary Table S1. Data collection and refinement statistics.

Supplementary Video S1. Soybean seeds inoculated with *B. subtilis* NBRC 16449 cells (supplied separately).

Supplementary Figure S1

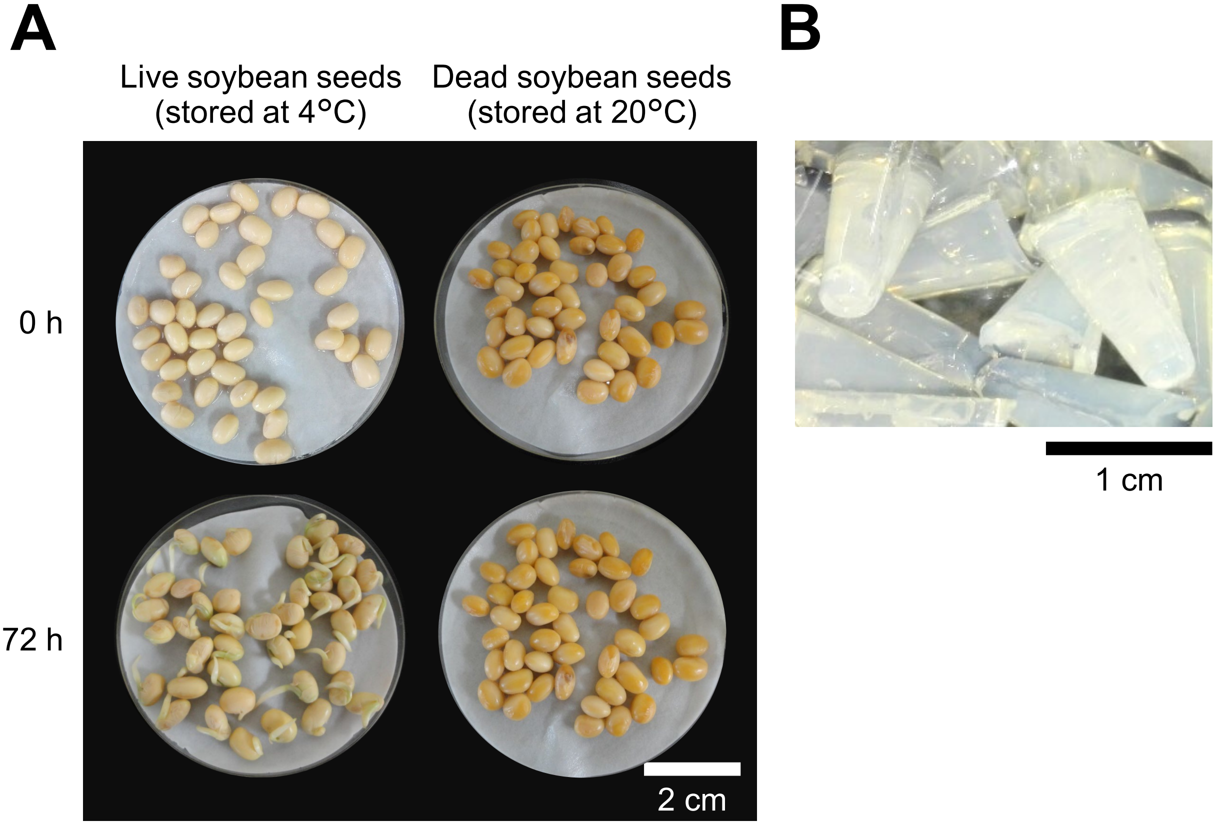

Supplementary Figure S1. Materials used for solid-state cultures in this study. (**A**) Live and dead soybean seeds. Commercially available soybean seeds were stored at 4°C (left) or at 20°C (right) for half a year. To test germination capacity, seeds were soaked in water and incubated on wet filter paper for 72 h. Germinating and non-germinating seeds were used as live and dead seeds, respectively. Bar, 2 cm. The profile of each plate is not an image cropped from different parts of the same plate or from different plates. (**B**) Soybean seed-mimicking LB sodium medium blocks. Bar, 1 cm.

Supplementary Figure S2

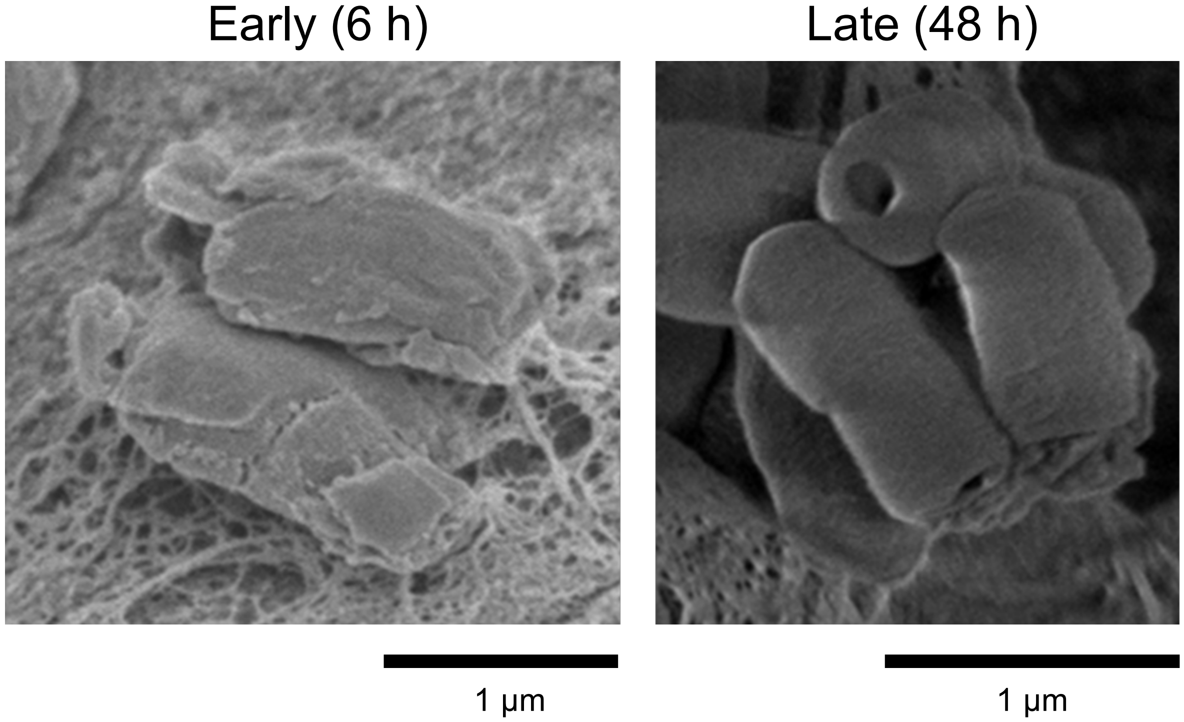

Supplementary Figure S2. SEM observation of *B. subtilis* NBRC 16449 cells on soybean-mimicking solid medium blocks. Bars, 1 µm.

Supplementary Figure S3

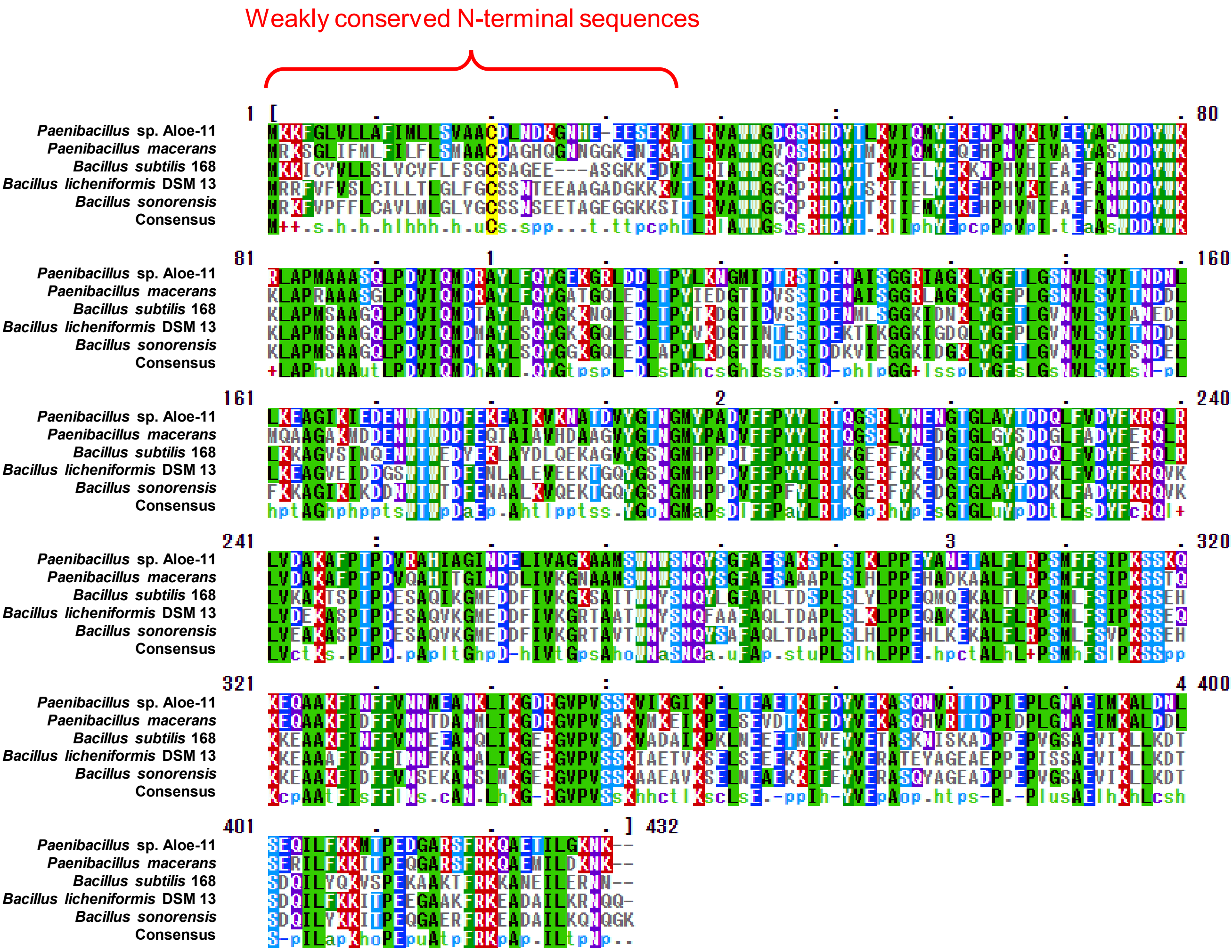

Supplementary Figure S3. Alignment of YesO orthologs. Amino acid sequences from *Bacillus subtilis* laboratory standard strain 168 and related species, *Bacillus sonorensis*, *Bacillus licheniformis* DSM 13, *Paenibacillus* sp. Aloe-11, and *Paenibacillus macerans*, were aligned using the Clustal Omega multiple sequence alignment program.

Supplementary Figure S4

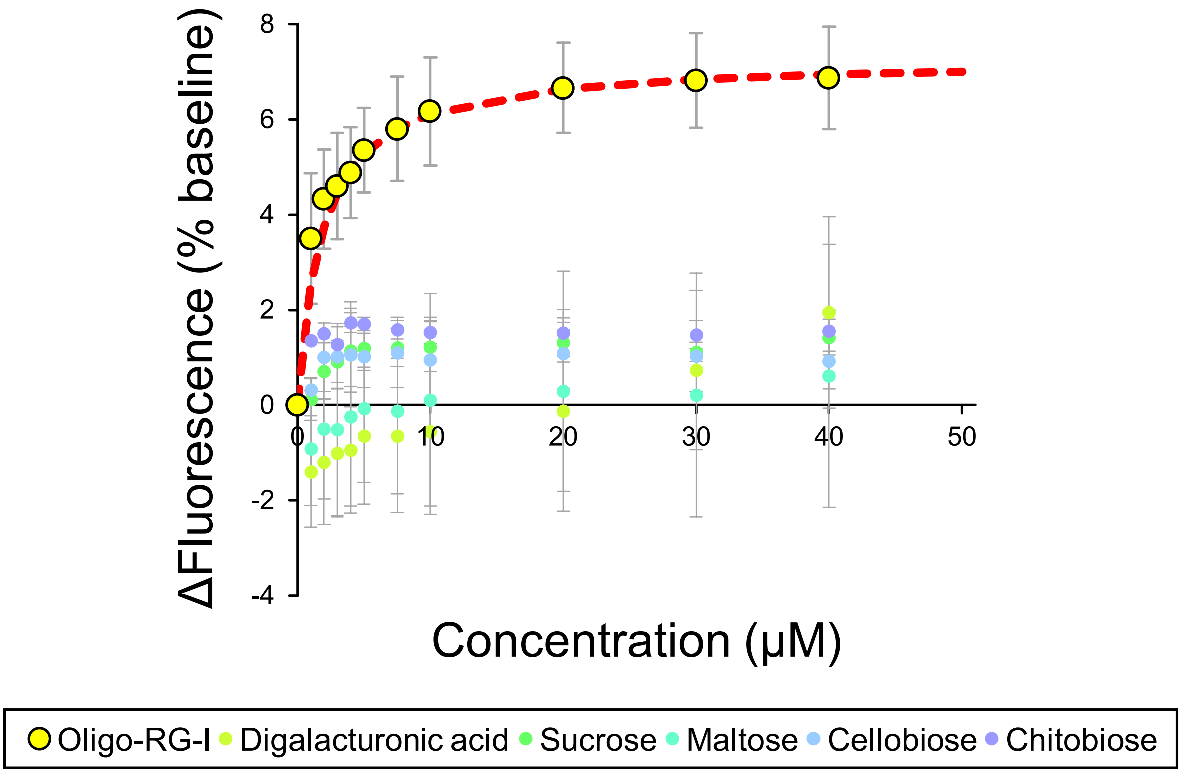

Supplementary Figure S4. Fluorescent spectrum analysis. Decreased fluorescent intensity (Δfluorescence) by increasing ligand concentrations was plotted after modification based on volume change in the cuvette. Each data represents the mean ± SD from three independent experiments.

Supplementary Figure S5

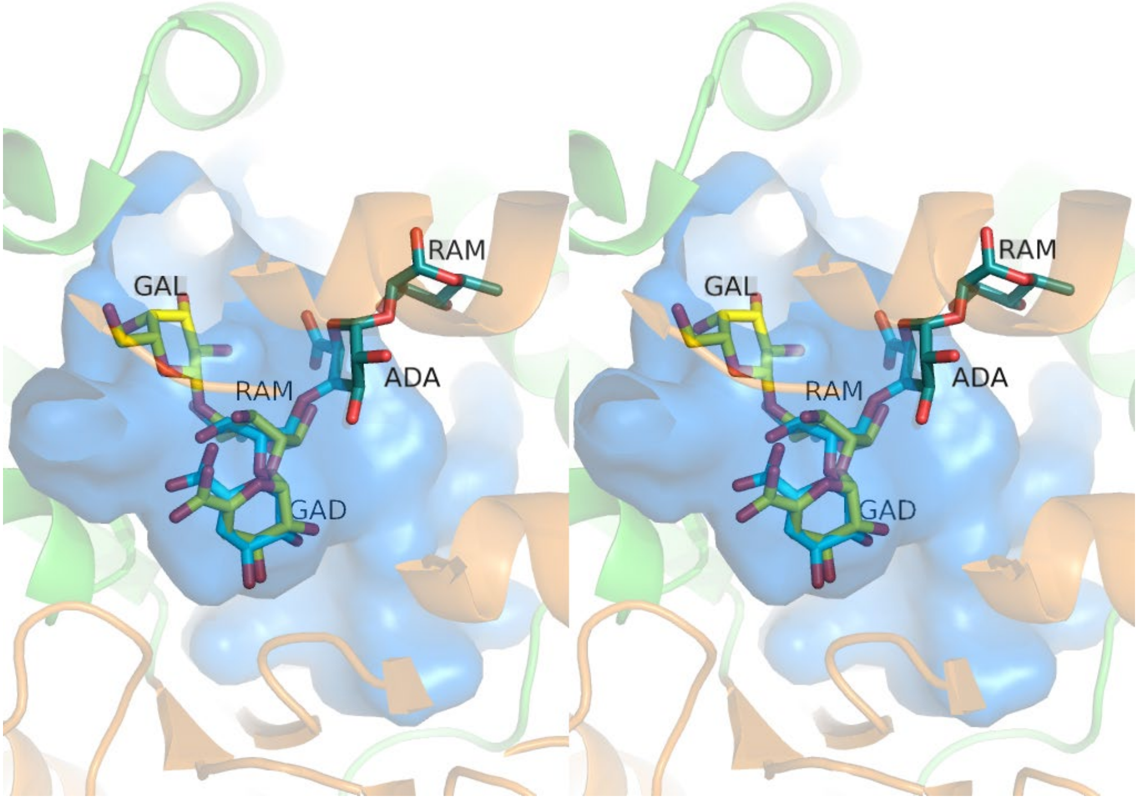

Supplementary Figure S5. The stereo diagram of the cavity of YesO/oligo-RG-I. The model contains cavity-bound RG-I trisaccharide (ΔGalUA-Rha-Gal; yellow) and pectin tetrasaccharide derived from PDB ID 5XQO (ΔGalUA-Rha-GalUA-Rha; cyan). GAD, unsaturated galacturonic acid; RAM, rhamnose; GAL, galactose; ADA, galacturonic acid. The cavity was calculated and drawn as blue surface by PyMOL (ver. 1.5). The ΔGalUA and Gal residues are located at the bottom and the entrance of the cavity, respectively. The ΔGalUA and Rha residues of tetrasaccharide are superimposed to the ΔGalUA and Rha residues of trisaccharide, suggesting that the two residues more strongly interact with the cavity than the Gal residue. The atoms of tetrasaccharide outside the cavity are colored dark cyan. Obviously, the whole fourth residue (Rha) and a part of the third residue (GalUA) of the tetrasaccharide are outside of the cavity, indicating that YesO may bind only disaccharide or trisaccharide derived from pectin digestion.

Supplementary Table S1

	YesO	YesO/oligo-RG-I
Space group	$P2_1$	$P2_1$
Unit cell parameters (Å, °)	a = 36.2, b = 108.6, c = 40.5<br>β = 101.6	a = 42.2, b = 78.6, c = 54.1<br>β = 102.0
**Data collection**		
Length (Å)	1.00	1.00
Resolution limit (Å)	50.0-1.97 (2.00-1.97)	50.0-1.58 (1.61-1.58)
Total reflections	92,100 (4,599)	223,681 (9,107)
Unique reflections	21,509 (1,095)	46,402 (2,118)
Redundancy	4.3 (4.2)	4.8 (4.3)
Completeness (%)	99.2 (99.5)	98.5 (90.4)
$I/\sigma(I)$	17.8 (4.52)	43.0 (6.41)
R_{merge} (%)	12.6 (34.1)	4.4 (24.4)
**Refinement**		
Model (residue/water/sugar)	379/116/0	396/126/3
Resolution limit (Å)	37.3-1.97 (2.06-1.97)	43.9-1.58 (1.62-1.58)
Used reflections	21,481 (2,556)	46,573 (2,823)
Completeness (%)	98.6 (94.0)	98.7 (96.0)
R-factor (%)	19.9 (19.9)	18.0 (20.3)
R_{free} (%)	25.8 (27.3)	20.4 (22.8)
**Root-mean-square deviation**		
Bond (Å)	0.007	0.006
Angle (°)	0.970	0.851
**Ramachandran plot (%)**		
Preferred regions	94.3	98.7
Allowed regions	5.7	1.3
Outliers regions	0.0	0.0

Supplementary Table S1. Data collection and refinement statistics. Data of the highest shells are given in parentheses.
